# Intestinal permeability and its relation to anthropometric and biochemical variables associated with cardiovascular risk in an elderly population

**DOI:** 10.1038/s41598-025-08045-8

**Published:** 2025-07-01

**Authors:** Maria Clara da Cruz Carvalho, Ana Carolina Costa Campos Mota, Daniele de Souza Marinho do Nascimento, Ingrid Naihara França de Sousa, Mariana Duarte Bona, Karla Danielly da Silva Ribeiro, Aldo Ângelo Moreira Lima, Bruna Leal Lima Maciel

**Affiliations:** 1 Post Graduate Program in Health Sciences, Center for Health Sciences, Federal University of Rio Grande do Norte, Natal, RN 59078-970 Brazil; 2 Post Graduate Program in Nutrition, Department of Nutrition, Federal University of Rio Grande do Norte, Natal, RN 59078-970 Brazil; 3 Department of Nutrition, Center for Health Science, Federal University of Rio Grande do Norte, Natal, RN 59078-970 Brazil; 4 Institute of Biomedicine, Department of Medicine, Federal University of Ceara, Fortaleza, 60430-270 Brazil

**Keywords:** Intestinal permeability, Elderly health, Vitamin A, Gastrointestinal diseases, Functional gastrointestinal disorders, Nutrition disorders

## Abstract

Aging reduces functional capacity, decreasing lean mass and immune function, possibly impacting the intestinal morphofunctional barrier. This study aimed to characterize intestinal permeability in an elderly population and its association with anthropometric and biochemical variables associated with cardiovascular risk. A cross-sectional study was conducted with 54 elderly individuals from Oct/19-Mar/23. Self-reported disease, anthropometric (weight, height, waist, and hip circumferences), and biochemical (lipid profile, glycemic, and serum retinol) data were collected. Intestinal permeability was assessed using the lactulose: mannitol (L: M) test, and stratified into percentile ≤ 50 or > 50. Diabetes, hypertension, and overweight were present in 25.9%, 53.7%, and 51.9% of the population, with no significant differences between those with L: M ≤ P50 or > P50. Median L: M was 0.037 (0.014; 0.060). Those with L: M > P50 had significantly lower levels of hip circumference [96.50 (93; 104) cm; *P* = 0.041] and serum retinol [0.95 (0.60; 1.16) mmol/L; *P* < 0.001], and these variables were also inversely associated with an odds for a L: M > P50 (AOR 0.93, 95% CI  0.86–0.99; *P* = 0.042; AOR 0.15, 95% CI  0.05–0.42; *P* < 0.001, respectively). We concluded that hip circumference and serum retinol were negatively associated with intestinal permeability in the studied elderly, and data indicate that lactulose and mannitol were positively associated with anthropometric and biochemical markers related to metabolic complications.

## Introduction

Aging is a complex process related to various changes in physiological, metabolic, and immune functions^1^. The World Health Organization (WHO) predicts that the number of individuals ≥ 60 years will triple by 2050^2^. Aging is associated with the development of various diseases that impair the quality of life and require increased healthcare needs, resulting in high costs to healthcare systems^3,4^.

Dysregulation and hyperactivation of inflammation are also observed during aging, leading to a chronic and persistent low-grade inflammatory state that correlates with the development of metabolic, cardiovascular, and intestinal diseases^5^. Thus, the functional decline of the immune system, a process known as immunosenescence, is a expected condition in the elderly and can negatively impact intestinal health^3,5^.

Hence, age is a factor that can cause modifications in the intestinal microbiota, inducing changes in intestinal permeability^5,6^. Intestinal permeability is a vital component of the intestinal functional barrier that regulates the passage of pro-inflammatory molecules, microorganisms, toxins, and antigens. Increased intestinal permeability allows the translocation of microorganisms from the intestinal lumen to the bloodstream, which may be related to the development of inflammatory diseases, intestinal disorders, diabetes, obesity, metabolic syndrome, cancer, and cardiovascular diseases^1,6,7^. Despite this assumption, studies evaluating intestinal permeability in the elderly are still limited and yield divergent results, as some have shown that this population may exhibit increased intestinal permeability, while others have not observed this association^8–10^.

Diet and its components play a fundamental role in maintaining intestinal health, as they directly influence the structure and function of the gut barrier. Among these dietary factors, retinoids play a key role in regulating the gene expression of intestinal epithelial barrier proteins, supporting mucosal function and protection. Cell-based studies and morphological evaluations in animal models have shown that vitamin A deficiency is linked to reduced villus height, shallower crypts, and lower expression of tight junction-related genes. As a result, insufficient vitamin A levels compromise the integrity of the intestinal epithelium, leading to increased permeability and a heightened risk of inflammation^11–13^. Although studies have shown an important effect of vitamin A in the intestinal barrier function in children^14–16^, there are no human studies considering elderly.

There are several techniques available to assess intestinal permeability, each offering different insights into gut barrier function. One widely used method involves measuring the urinary excretion of probe molecules, such as in sugar absorption tests like the lactulose: mannitol ratio, which assess the extent to which different-sized molecules can cross the intestinal barrier. Another common approach is the evaluation of circulating biomarkers associated with mucosal damage, including zonulin and lipopolysaccharide (LPS). In addition, in vitro analyses using cell lines or human biopsies can be used to study the expression of tight junction proteins, such as claudins, occludins, and zonula occludens. Endoscopic techniques also represent valuable tools for assessing intestinal permeability in clinical settings^17^. Although these techniques are promising, the lactulose: mannitol test allows the assessment of both transcellular (%mannitol) and paracellular absorption (%lactulose), especially in the small intestine, where most of nutrient’s digestion and absorption occur^17^, enriching the analysis of the intestinal barrier permeability function and possible variables associated with both transcellular and paracellular absorption.

Therefore, considering that changes in intestinal permeability may be related to impacts on the health of the elderly and the increasing growth of this population, it is important to understand intestinal permeability in the elderly. Thus, the objective of the study was to characterize intestinal permeability in elderly individuals and determine its associations with anthropometric and biochemical variables associated with cardiovascular risk. Considering the evidence, the hypothesis under study is that elderly with worse anthropometric and biochemical parameters present higher intestinal permeability.

## Materials and methods

### Ethical considerations

All methods were carried out in accordance with relevant guidelines and regulations. The present study was submitted and formally approved to the Research Ethics Committee (CEP) involving human subjects of the Onofre Lopes University Hospital at the Federal University of Rio Grande do Norte - HUOL/UFRN (Certificate of Presentation for Ethical Appreciation (CAAE) 18923719000005292; number 3623997). All individuals eligible for the research were informed about the objectives, risks, and benefits, and those who agreed and signed the Informed Consent Form, participated in the study.

### Study population and data collection

This is a cross-sectional study conducted in Natal, Rio Grande do Norte, Brazil, with data collection occurring in two periods: from October 2019 to March 2020 and from November 2021 to March 2023. The first period was interrupted due to the COVID-19 pandemic. During this first period, data collection occurred at the participants’ homes. The second period of the study, due to social isolation restrictions, was conducted at the Department of Nutrition in UFRN. Data collection followed the same standards as in the first period. All biosafety measures were implemented. Throughout both periods, the study was conducted by properly trained professionals.

It was estimated by the Pearson or Spearman correlation test of 0.40, that a sample size of 53 individuals was necessary to achieve 80% power and an alpha of 95% (0.05), with an estimated drop-out rate of 10%^18^. The recruitment was performed by promotion on social media of the research and telephone contact. Individuals with any cognitive deficits unable to respond to questionnaires, individuals using antibiotics, antimicrobials, undergoing chemotherapy and/or radiotherapy, with digestive diseases, or presenting any type of intestinal infection, diarrhea, vomiting, or fever were excluded from the study.

In total, 66 individuals were recruited to participate in the study. Of these, 11 participants were excluded due to digestive disease presentation, and 1 due to the impossibility of blood and urine collection. In the end, 54 individuals participated in the study.

### Anthropometric assessment

Measurements of weight, height, waist circumference, and hip circumference were collected by trained professionals. Body weight was measured using an electronic anthropometric scale with a capacity of 150 kg, and height was measured using a fixed stadiometer with a precision of 1.0 mm. Participants were barefoot and wore light clothing without accessories, instructed to face the evaluator with their head aligned with the Frankfurt plane. Body Mass Index (BMI) was calculated using the formula BMI = Weight (Kg)/Height(m)^2^ and classified according to reference values defined by Lipschitz, 1994^19^. Waist circumference measurement was taken using an ergonomic tape measure, with the individual in an upright position, encircling the midpoint between the last rib and the iliac crest^20,21^. Hip circumference was measured by encircling the largest gluteal protuberance with the individual in the same position mentioned earlier. These measurements were used to calculate the Waist-to-Hip ratio (WHR). The cutoff values recommended by the world health organization^22^ was used as reference points.

### Intestinal barrier assessment

All study participants were given an oral solution containing 5.0 g of lactulose and 1.0 g of mannitol, dissolved in 20 mL of water^23^. Participants were required to fast for a minimum of 2 h and were instructed to empty their bladder before ingesting the oral solution. After ingesting the solution, participants remained fasting for an additional 1 h. Urine volume was collected over a period of 5 h. To preserve urine volume, 1 drop of 2.35% chlorhexidine was added to every 50 mL of collected urine. Urine volumes were recorded and stored at -80ºC until the day of analysis. Analyses were conducted using high-performance liquid chromatography with pulsed amperometric detection (HPLC-PAD), following Barboza et al. (1999) methodology^24^.

### Determination of vitamin A levels

Blood samples were collected by venipuncture by trained professionals. These samples were placed into tubes for serology and, upon thawing, subjected to centrifugation at room temperature for 5 min (500 x g) to separate whole blood and serum components. Subsequently, aliquots containing 500 µl of serum were stored at − 80 °C until biochemical analysis. The extraction of retinol serum followed the adapted method of Ortega et al.^25^ and was analyzed using High-Performance Liquid Chromatography (HPLC) (Shimadzu, Kyoto, Japan) at a wavelength of 325 nm. Retinol levels were calculated in mmol/L, with levels below 0.7 µmol/L (20 µg/dL) considered low according to WHO recommendations^26^.

### Evaluation of biochemical parameters

For the assessment of biochemical parameters, 5 mL of blood were collected via venipuncture by a trained and outsourced nursing technician in the same day of the lactulose: mannitol test. The collection took place in the morning, with participants fasting for 8 h. The samples were collected in tubes without anticoagulant but with separator gel and then sent for analysis of total cholesterol and fractions, triglycerides, glucose, insulin, and ultrasensitive C-reactive protein (Us-CRP) at a reference laboratory in the municipality of Natal-RN, contracted for this purpose. Enzymatic techniques were employed to assess fasting blood glucose, total cholesterol, and triglycerides. High-density lipoprotein cholesterol (HDL-c) concentrations were determined using a homogeneous enzymatic colorimetric assay. Low-density lipoprotein cholesterol (LDL-c) levels were estimated through the Friedewald equation [LDL-c = Total cholesterol – HDL-c + (Triglycerides/5)]. Insulin levels were quantified via a sandwich-based immunoassay, while ultrasensitive C-reactive protein (Us-CRP) was measured using an immunoturbidimetric method. All evaluations were conducted through an automated system (COBAS 6000-Roche^®^ Professional Diagnostics, Risch-Rotkreuz, Switzerland).

### Statistical analysis

The collected data were analyzed using the Statistical Package for the Social Sciences (IBM SPSS Statistics 26). The Kolmogorov-Smirnov test was used to determine the normality of the variables. The continuous variables were represented as median (Q1–Q3) or median (SD) and categorical variables by percentage and absolute n. To evaluate the differences between the continuous variables, we used the Student’s T test or the Mann–Whitney’s test, depending on the nature of the variables. The Chi-square test was used to detect the association between groups of categorical variables. Spearman’s correlation was used to assess the correlations between permeability variables (lactulose, mannitol, and the L: M ratio) and anthropometric and biochemical variables. *P* values < 0.05 were considered statistically significant.

The variables that showed correlation with the L: M ratio in the Spearman test were used for logistic regression models, first exploring the effect of a single variable on intestinal permeability, and their unadjusted odds ratios (OR) and respective 95% confidence intervals (95% CI) were demonstrated. Then, adjusted logistic regression models were calculated, considering the dichotomized L: M ratio classification as a dependent variable (1 = > P50, 0 = ≤ P50). The adjustment of the final model shown was guaranteed by observing the Omnibus test, with p values less than 0.05, and the Hosmer and Lemeshow test, considering p values greater than 0.05. *n* = 15 was considered for each independent variable used in the model. Thus, retinol levels, age and hip circumference were included in the final model as independent variables. The adjusted odds ratios (AOR) and their respective 95% CI were presented.

## Results

The studied sample of the elderly population comprised 54 individuals, who were stratified into groups based on percentiles ≤ P50 or > P50 according to the lactulose: mannitol ratio. Characterization analysis revealed no significant differences between groups with higher or lower intestinal permeability concerning socio-economic and biochemical profiles (Table 1). A higher hip circumference was observed in elderly individuals with lower permeability, with a median of 105.50 (95; 116) cm (*P* value = 0.041).

**Table 1:** Characterization of the studied population according to the stratification of the lactulose: mannitol ratio.

Variables	L: M ratio		*P* value
	≤ P50 *n* = 27	> P50 *n* = 27	
Socioeconomic profile			
Income (BRL), median (Q1–Q3)	3647 (2500; 11,000)	5000 (2000; 8000)	0.938^a^
Age (years), mean (SD)	68 (6)	69 (7)	0.460^b^
Gender, n (%)			0.580^c^
Female	17 (63%)	15 (55.6%)	
Male	10 (37%)	12 (44.4%)	
Race, n (%)			0.486^c^
White	14 (51.9%)	12 (44.4%)	
Mixed race	10 (37%)	10 (37%)	
Black	3 (11.11%)	5 (18.5%)	
Education, n (%)			0.269^c^
None	2 (7.4%)	1 (3.7%)	
Incomplete elementary school	1 (3.7%)	3 (11.11%)	
Complete elementary school	6 (22.2%)	2 (7.4%)	
Incomplete high school	3 (11.1%)	-	
Complete high school	6 (22.2%)	7 (25.9%)	
Complete technical course	3.7% (1)	1 (3.7%)	
Incomplete college	–	–	
Complete college	8 (29.6%)	13 (48.1%)	
Physical activity practice, n (%)			0.580^c^
Yes	10 (37%)	12 (44.4%)	
No	17 (63%)	15 (55.6%)	
Smoking, n (%)			-
Yes	–	–	
No	27 (100%)	27 (100%)	
Alcohol consumption, n (%)			0.761^c^
Yes	8 (29.6%)	7 (25.9%)	
No	19 (70.4%)	20 (74.1%)	
Anthropometric variables			
BMI (kg/m^2^), median (Q1–Q3)	28.40 (24.54; 33.90)	25.21 (22.75; 29.85)	0.050^a^
Nutritional status, n (%)			0.137^c^
Low weight	2 (7.4%)	5 (18.5%)	
Eutrophic	9 (33.3%)	13 (48.1%)	
Overweight	16 (59.3%)	9 (33.3%)	
Weight (kg), mean (SD)	75.85 (15.35)	68.82 (15.56)	0.261^b^
Waist-to-hip ratio, mean (SD)	0.93 (0.07)	0.95 (0.07)	0.221^b^
Waist Circumference (cm), mean (SD)	97.73 (14.32)	93.97 (9.65)	0.318^b^
Hip Circumference, median (Q1 – Q3)	105.50 (95; 116)	96.50 (93; 104)	**0.041** ^**a**^
Biochemical variables			
Triglycerides (mg/dL), median (Q1–Q3)	128 (93; 215)	140 (97; 174)	0.653^a^
HDL-c (mg/dL), mean (SD)	51 (13)	50 (13)	0.702^b^
LDL-c (mg/dL), mean (SD)	128.2 (33.7)	117.8 (47.1)	0.408^b^
VLDL-c (mg/dL), median (Q1–Q3)	25.40 (18.60; 39.80)	28 (19.40; 34.80)	0.852^a^
Total Cholesterol (mg/dL), mean (SD)	209 (37)	195 (54)	0.337^b^
HOMA-IR, median (Q1–Q3)	2.45 (1.44; 6.16)	2.40 (1.54; 4.14)	0.887^a^
HOMA-B, median (Q1–Q3)	127.60 (94.30; 173.40)	101.20 (72.30; 152.80)	0.210^a^
Glucose (mg/dL), median (Q1–Q3)	96 (86; 115)	99 (89; 108)	0.315^a^
Insulin (mU/dL), median (Q1–Q3)	11 (6.03; 19.05)	9 (7; 16)	0.723^a^
Us-CRP (mg/dL), median (Q1–Q3)	0.15 (0.07; 0,31)	0.16 (0.13; 0.33)	0.299^a^
Chronic diseases, n (%)			
Depression	5 (18.5%)	1 (3.7%)	0.083^c^
Migraine	4 (14.8%)	1 (3.7%)	0.159^c^
Sinusitis	4 (14.8%)	3 (11.1%)	0.685^c^
Lung disease	2 (7.4%)	2 (7.4%)	1.000^c^
Hypertension	16 (59.3%)	13 (48.1%)	0.413^c^
Diabetes	5 (18.5%)	9 (33.3%)	0.214^c^

BMI, Body Mass Index; kg/m^2^, kilograms (per square meter); cm, centimeters. TGL, Triglycerides; HDL-c, high-density lipoprotein cholesterol; LDL-c, low-density lipoprotein cholesterol; VLDL-c, very low-density lipoprotein cholesterol; HOMA-IR, homeostasis model assessment of insulin resistance; HOMA-B, homeostasis model assessment of beta-cell function; Us-CRP, ultrasensitive C-reactive protein. mg/dL, milligrams per deciliter; mU/dL, milliequivalents per deciliter. Continuous variables were represented by mean (standard deviation) or median (IQR), and categorical variables by n (percentage). ^a^Mann–Whitney’s test was used to evaluate the difference between medians, ^b^Student’s T test was used to assess the difference between means, and ^c^Chi-square test was used to compare categorical variables. *P* values < 0.05 were considered statistically significant.

Lower levels of vitamin A [0.95 (0.60; 1.16); *P* < 0.001] and higher deficiency in this nutrient [32%; *P* = 0.007] were identified in elderly individuals with increased intestinal permeability (Table 2).

**Table 2:** Retinol serum concentrations and retinol status, considering intestinal permeability in the studied population.

Variables	L: M ratio		*P* value
	≤ P50 *n* = 27	> P50 *n* = 27	
Vitamin A (µmol/L)	2.06 (1.34; 2.40)	0.95 (0.60; 1.16)	**< 0.001**
Vitamin A, status, n (%)			
Adequate	26 (96.3%)	17 (68%)	**0.007**
Deficiency	1 (3.7%)	8 (32%)	

Continuous variables were represented by median (Q1; Q3), and categorical variables by absolute n (percentage). µmol/L, micromoles per liter; µg/dL; micrograms per deciliter. Continuous variables were represented by median (IQR), and categorical variables by n (percentage). The Chi-square test was used to compare categorical variables, and the Mann–Whitney’s test was used to evaluate the difference between medians. *P values* < 0.05 were considered statistically significant.

Regarding markers of intestinal permeability, the L: M ratio was negatively correlated with serum retinol levels (ρ = − 0.510; *P* < 0.001) and hip circumference (ρ = − 0.286; *P* = 0.036). Conversely, lactulose was negatively correlated with serum retinol levels (ρ = − 0.529; *P* < 0.001) and positively correlated with blood glucose levels (ρ = 0.351; *P* = 0.009). Mannitol was positively associated with anthropometric parameters: waist circumference, waist-to-hip ratio, and BMI (ρ = 0.283; *P* = 0.038; ρ = 0.351; *P* = 0.009; ρ = 0.270; *P* = 0.048; respectively), and biochemical parameters: triglycerides, insulin, glucose, HOMA-IR, VLDL-c (ρ = 0.384; *P* = 0.004; ρ = 0.389; *P* = 0.004; ρ = 0.453; *P* = 0.001; ρ = 0.390; *P* = 0.004; ρ = 0.367; *P* = 0.007; respectively). Mannitol was negatively correlated with HDL-c levels (ρ = − 0.309; *P* = 0.023), as shown in Table 3.

As presented in Table 4, the regression model demonstrated that both hip circumference and serum retinol levels were inversely associated with the odds of having and L: M > P50 (AOR  0.93; 95% CI 0.86–0.99; *P* = 0.042; AOR  0.15; 95% CI  0.05–0.42; *P* < 0.0005).

**Table 3:** Spearman correlations (ρ) between lactulose: mannitol ratio and anthropometric and biochemical variables in the studied population.

Anthropometric variables	L: M ratio	%L	%M
BMI (kg/m^2^)	− 0.221	− 0.070	0.270*
Waist circumference (cm)	− 0.127	0.022	0.283*
Hip circumference (cm)	− 0.286*	− 0.166	0.152
Waist-to-hip ratio	0.086	0.207	0.351**
Biochemical variables	L: M ratio	%L	%M
Total cholesterol (mg/dL)	− 0.059	− 0.042	− 0.035
TGL (mg/dL)	− 0.027	0.142	0.384**
HDL-c (mg/dL)	0.023	− 0.053	− 0.309*
LDL-c (mg/dL)	− 0.080	− 0.106	− 0.068
VLDL-c (mg/dL)	0.030	0.210	0.367**
HOMA-IR	0.061	0.255	0.390**
HOMA-B	− 0.150	− 0.081	0.058
Fasting glucose (mg/dL)	0.164	0.351**	0.453**
Fasting insulin (µU/mL)	− 0.050	0.126	0.389**
Us-CRP (mg/dL)	0.176	0.223	0.079
Retinol (µmol/L)	− 0.510**	− 0.529**	0.061

BMI, Body Mass Index; kg/m^2^, kilograms per meter squared; cm, centimeters; mg/dL, milligrams per deciliter; TGL, triglycerides; HDL-c, high density lipoprotein cholesterol; LDL-c, low-density lipoprotein cholesterol; VLDL-c, very low-density lipoprotein cholesterol; HOMA-IR, homeostasis assessment model of insulin resistance; HOMA-B, homeostasis assessment model of beta-cell function; Us-CRP, ultrasensitive C-reactive protein. mg/dL, milligrams per deciliter. *P value < 0.05 and **P value < 0.01 were considered statistically significant and are in bold in the table.

**Table 4:** Logistic regression for variables associated with a lactulose: mannitol (L: M) ratio > P50.

Variables	L: M > P50			
	OR (95% CI)	*P* value	AOR (95% CI)	*P* value
Retinol, µmol/L	0.17 (0.06–0.45)	**< 0.001**	0.15 (0.05–0.42)	**< 0.001**
Hip circumference, cm	0.95 (0.89–1.00)	0.058	0.93 (0.87–0.99)	**0.042**
Age, years	0.88 (0.92–1.09)	0.879	0.99 (0.87–1.14)	0.932

OR, odds ratio; AOR, adjusted odds ratio; 95% CI, 95% confidence interval (lower limit - upper limit). *P* values < 0.05 were considered statistically significant and are highlighted in bold.

## Discussion

In this cross-sectional study, we investigated the association of intestinal permeability with biochemical and anthropometric variables in elderly. In this population, increased permeability was associated with decreased serum retinol levels and hip circumference. Lactulose and mannitol were also positively associated with anthropometric and biochemical markers related to metabolic complications. These findings underscore the importance of anthropometric and biochemical profiles, specially adequate vitamin A status in maintaining intestinal barrier integrity.

The assessment of intestinal permeability is often determined using the lactulose: mannitol test, based on the analysis of urinary excretion. This test is widely recognized and utilized for evaluating intestinal permeability in humans, primarily due to its non-invasive nature, relatively low cost, and the ability of lactulose and mannitol to be absorbed passively, without undergoing metabolism and being excreted in urine^27^. However, the association between changes in intestinal permeability and the aging process are controversial^17^.

Studies have suggested that increased intestinal permeability is associated with aging, leading to increased bacterial translocation and resulting in elevated systemic inflammation, thus contributing to the development and progression of diseases^28–31^, including metabolic syndromes, reduced physical capacity, and mortality^7^. On the other hand, there is also evidence demonstrating a slight decrease in the L: M ratio in the elderly, but not significantly different compared to adults or with age progression^31,32^. In our study, we did not find significant difference in age, considering lower or higher intestinal permeability in the studied elderly.

The absorption of mannitol and lactulose reflects transcellular and paracellular permeability in the small intestine, respectively, indicating damage to mucosal cells and tight junctions when associated with decreased mannitol absorption and increased lactulose absorption. We found an inverse correlation of the L: M ratio and lactulose with serum retinol levels; thus, higher permeability and damage to tight junctions were associated with lower circulating vitamin A levels. Hip circumference showed an inverse correlation with the L: M ratio, which contrasts with the findings by Di Palo et al.^33^. Lactulose correlated positively with blood glucose, while mannitol correlated with anthropometric parameters (waist circumference, waist-to-hip ratio, BMI) and biochemical markers (triglycerides, insulin, glucose, HOMA-IR, VLDL-c). Similar results were reported by Del Bo’ et al.^6^, who, after a dietary intervention with polyphenols, demonstrated lower serum levels of plasma zonulin in elderly subjects with higher initial BMI and HOMA-IR (indicating glucose and insulin resistance), suggesting a relationship between intestinal permeability and metabolic disorders. Di Palo et al.^33^, on the other hand, found a positive correlation between the L: M ratio and waist circumference and BMI, consistent with our findings. In the present study positive correlation was found between the %mannitol excretion and BMI (ρ = 0.270; *P* < 0.05), waist circumference (ρ = 0.283; *P* < 0.05), and waist-to-hip ratio (ρ = 0.351; *P* < 0.01). What may help explain the results found in our study is that a larger intestinal epithelial surface area is observed in individuals with higher weight and abdominal circumference, which may lead to increased mannitol excretion and, consequently, greater permeability^34^.

Patients with higher BMI often exhibit low-grade inflammation and systemic inflammation. During the inflammatory process, changes in intestinal barrier homeostasis occur, impairing permeability, leading to colonic dysbiosis. Moreover, aging itself alters this intestinal microbiome^7^. This microbiome plays crucial roles in producing vitamins, biotransforming nutrients, and generating waste products^33^ and is involved in numerous metabolic reactions that influence normal physiology and metabolism^9^. Depending on the composition of this microbiome and the level of intestinal permeability, there can be alterations in both nutrient absorption and endogenous production, potentially resulting in systemic reductions in vitamin A serum levels, as observed in our study.

In the regression analysis, an inverse association was demonstrated between retinol levels and intestinal permeability (AOR  0.15; 95% CI 0.05–0.42; *P* < 0.001). In this context, elderly individuals with higher intestinal permeability exhibited lower retinol values, with a median of 0.95 (0.60; 1.16) µmol/L. Vitamin A is a fat-soluble molecule that belongs to the group of retinoids with biological activity. It is recognized as a key nutrient in maintaining intestinal health homeostasis, associated with the regulation and differentiation of components in both innate and adaptive immune processes, crucial for balancing immunogenicity and tolerance at the intestinal barrier^17^. Morphological analyses in animal models have shown that low levels of vitamin A are associated with reduced villus height, decreased crypt depth, and lower gene expression levels of tight junction markers^16,18^. Thus, low levels of vitamin A compromise epithelial integrity, potentially leading to increased intestinal permeability and inflammation. Randomized clinical trials have shown that vitamin A supplementation in children significantly improved response to infections by promoting recovery of intestinal barrier integrity^14–16^. As far as we know, this is the first study to evaluate the association between vitamin A levels and intestinal permeability in elderly population, without infection.

The present study has some limitations. Although other markers such as serum zonulin could have been considered, we focused on evaluating the L: M test, which is considered a sensitive and specific tool for assessing intestinal permeability, allowing for paracellular and transcellular permeation. Thus, it is a low-cost, simple, non-invasive method, and can effectively detect changes in permeability^35^. This is relevant due to potential complications in urinary function among older adults, which can arise from various factors including bladder issues, incontinence, medication use, and low fluid intake — common in this age group^36^. Nevertheless, all participants were carefully monitored by trained professionals during data collection and none declared renal diseases. Additionally, only individuals with adequate motor capacity were recruited to perform the test, aiming to minimize potential biases. Furthermore, analysis of intestinal microbiota and assessment of diet were not conducted, factors that could help explain the findings. The fact that questions related to socioeconomic profile—such as physical activity, smoking status, and alcohol consumption—were self-reported constitutes a limitation of the study, as they may be subjected to recall bias and social desirability bias.

Nonetheless, the associations found make significant contributions to establishing the relationship between intestinal permeability and age, providing insights for conducting more comprehensive studies that include these variables. To our knowledge, this is the first study to investigate the association between vitamin A levels and intestinal permeability in elderly. Exploring the role of diet and its components, and their relationship with changes in intestinal permeability in elderly population, may help elucidate these differences and should be investigated in future research.

## Conclusion

Hip circumference and serum retinol were inversely associated with higher intestinal permeability in elderly, indicating that these variables should be monitored to improve intestinal permeability in the studied elderly population. Conversely, lactulose and mannitol revealed a positive association with anthropometric and biochemical markers related to metabolic complications.

## Data Availability

The datasets analyzed during the current study are available from the corresponding author on reasonable request.
